# The influence of summer closure on serious postoperative complications in bariatric surgery

**DOI:** 10.1007/s00423-022-02566-w

**Published:** 2022-06-02

**Authors:** Johanna Fall, Magnus Sundbom, Erik Stenberg

**Affiliations:** 1 Department of Surgery, Faculty of Medicine and Health, Örebro University, Örebro, Sweden; 2 Department of Surgical Sciences, Uppsala University, Uppsala, Sweden; 3 Department of Surgery, Örebro University Hospital, 701 82 Örebro, Sweden

**Keywords:** Humans, Gastric bypass, Sleeve gastrectomy, Laparoscopy, Obesity, Postoperative complication

## Abstract

**Introduction:**

Bariatric surgery is an effective method of treating obesity, with gastric bypass and sleeve gastrectomy being the most common techniques used worldwide. Despite the technical challenges in these methods, little is known about the effects of summer closure on the incidence of serious postoperative complications in surgeries performed shortly after summer vacation. This has therefore been studied in our large cohort.

**Materials and methods:**

A retrospective cohort study based on data from the Scandinavian Obesity Surgery Registry was conducted. Patients who underwent a primary gastric bypass or sleeve gastrectomy operation between 2010 and 2019 were included. The rate of serious complications within 30 days after surgery for patients who underwent surgery the first month after summer closure was compared to those who underwent surgery during the rest of the year using the *χ*^2^ test and adjusted logistic regression.

**Results:**

The study included 42,404 patients, 36,094 of whom underwent gastric bypass and 6310 of whom received sleeve gastrectomy. Summer closure was associated with an increased risk for serious postoperative complications in gastric bypass surgery (adjusted odds ratio (adj-OR) = 1.17; 95% confidence interval (CI): 1.01–1.36). No statistically significant association was seen for sleeve gastrectomy (adj-OR = 1.17; 95% CI: 0.72–1.91), nor in overall complication rate.

**Conclusions:**

Summer closure increases the risk of serious postoperative complications in gastric bypass surgery. No statistically significant association was found for sleeve gastrectomy surgery.

**Supplementary Information:**

The online version contains supplementary material available at 10.1007/s00423-022-02566-w.

## Introduction

Bariatric surgery is currently the most effective treatment for obesity, resulting in long-term weight reduction and the resolution of metabolic comorbidities for many patients [1, 2], with Roux-en-Y gastric bypass (RYGB) and sleeve gastrectomy (SG) being the most common types of bariatric surgical procedures performed worldwide [3].

As with any type of surgery, bariatric surgery entails a risk of postoperative complications. Although the introduction of the laparoscopic technique has reduced the overall complication rate, the patient is still at risk of serious adverse postoperative events such as leakage, bleeding, or small bowel obstruction [4–6].

Several studies have investigated whether there is an association between the day, week, or season of surgery and outcomes. In a large cohort of patients, the mortality rate after surgery starting in the afternoon was higher than that after surgery starting in the morning, regardless of the surgical discipline and the urgency of the performed surgery. No difference in mortality between weekends and weekdays was found [7]. For bariatric surgery, seasonality in complications with an increased risk of sepsis and deep vein thrombosis (DVT) in the colder season compared to the summer season has been reported [8]. Higher complication rates during the first weeks when restarting bariatric surgery after summer vacation have been observed as a personal experience, but to the best of our knowledge, the effect of summer closure of bariatric surgery centers on postoperative adverse events has not been studied previously.

Therefore, the aim of this study was to investigate whether there is an increased risk of serious postoperative complications within the 30-day postoperative period in patients who underwent RYGB or SG surgery during the start-up after summer closure.

## Material and methods

This was a retrospective cohort study based on prospectively collected data extracted from the Scandinavian Obesity Surgery Registry (SOReg). The SOReg, established in 2007, is a national research and quality registry, where individual data for patients undergoing bariatric surgery are continuously collected as part of clinical practice [9]. All Swedish centers for bariatric surgery use the registry and a recent audit showed that it covers > 97% of all bariatric procedures in Sweden [10]. The SOReg contains patient demographic data, information on comorbid diseases (sleep apnea, hypertension, diabetes, dyslipidemia, dyspepsia, and depression), and perioperative and postoperative data [10]. Comorbidities are defined as a specified condition requiring pharmacological treatment or treatment with positive airway pressure (sleep apnea) [9]

During the study period, the majority of bariatric surgical procedures were performed within the public healthcare system, financed by public means. The majority of operations were performed by specialists in bariatric surgery or specialists in upper GI surgery under direct supervision of a bariatric surgeon. Most healthcare providers working in the publicly funded healthcare sector go on vacation for at least 4 weeks during summer.

Patients who underwent primary laparoscopic RYGB or SG surgery between 2010 and 2019 were eligible for inclusion in the study. For each facility having entered data in SOReg, it was investigated whether there was a gap of at least four consecutive weeks with no registered elective bariatric surgical procedure during the summer months of June through August. If a gap was not found, all patients who underwent surgery at that facility that year were excluded from the study. Patients who underwent surgery during the first 4 weeks after summer closure were compared to those who underwent surgery during the rest of the year.

### Outcomes

The primary outcome was serious complications within 30 days of surgery. Secondary outcomes were duration of surgery, duration of postoperative hospital stay, readmission, and the occurrence of specified postoperative complications (leak, bleeding, abscess, small bowel obstruction, DVT, pulmonary complication, cardiovascular complication, wound complication, stricture, marginal ulcer, urinary tract infection).

### Definitions

Postoperative complications were graded according to the Clavien-Dindo classification system [11]. A complication graded as Clavien-Dindo score ≥ 3b, i.e., a complication requiring intervention under general anesthesia, resulting in single or multiorgan failure, or death, was considered a serious postoperative complication [11]. Annual surgical volume at each center was classified as low-volume (< 100 bariatric surgical operations/year), mid-volume (100–249 operations/year), and high-volume (≥ 250 operations/year) [12].

### Statistical analysis

Baseline characteristics were presented as means ± standard deviations or numbers (percentages) as appropriate with comparison across groups using the *χ*^2^ test for categorical variables and the *t* test of means for continuous variables. Outcomes were analyzed with stratification by surgical method. The *χ*^2^ test and adjusted logistic regression (adjusted for sex, age, BMI, comorbidities, concurrent surgery, and year of surgery) were performed for the primary outcome and all binary secondary outcomes. The duration of surgery was analyzed using unadjusted and adjusted linear regression (adjusted for sex, age, BMI, comorbidities, concurrent surgery, and year of surgery). Due to a nonnormal distribution, the duration of postoperative hospital stay was analyzed with the Mann–Whitney *U* test, and the effect size was calculated with eta squared. A value of *p* < 0.05 was considered to represent statistical significance. Relative risks (RRs), odds ratios (ORs), and beta values were calculated with a 95% confidence interval (CI). The analyses were considered exploratory and as such no adjustment for multiple comparisons was performed. Missing data was handled by listwise deletion. A post hoc sensitivity analysis was performed including patients lost to follow-up. In this analysis, logistic regression was run twice, once with a configuration assuming that all patients lost to follow-up had a serious complication and once assuming no patient had a serious complication. A further post hoc sensitivity analysis compared the first 4 weeks after summer with the remaining part of the year for patients operated at centers not closing during summer. IBM SPSS Statistics version 22 was used for all statistical analyses.

## Results

During the study period, 61,786 patients were identified from the SOReg. Of these, 17,683 did not meet the inclusion criteria because the surgery center did not close during summer that year, and an additional 1699 (3.9%) patients were lost to follow-up at 30 days. Of the 42,404 remaining patients, 36,094 underwent RYGB surgery and 6310 underwent SG. Concerning hospital volume, 21%, 46%, and 33% of the operations were performed at low-, mid-, and high-volume centers.

In the RYGB group, 74.9% of patients were women and the mean age at surgery was 41.0 ± 11.3 years. Patients operated on after summer closure were more often women, who had slightly lower BMIs and less often had concurrent surgery. The corresponding figures for the SG group were 78.4% female patients and a mean age of 41.1 ± 11.1 years with statistically significant differences only in the concurrent surgery variable (Table 1).

**Table 1:** Baseline characteristics of patients receiving gastric bypass or sleeve gastrectomy within 4 weeks after summer closure and those having surgery during the remaining part of the year

	Gastric bypass		Sleeve gastrectomy	
	After the summer closure	Remaining part the of year	After summer the closure	Remaining part the of year
Number of patients, *n*	5462	30,632	912	5398
Female sex, *n* (%)	4152 (76.0)*	22,888 (74.7)*	710 (77.9)	4234 (78.4)
Age at surgery, mean ± SD, yrs	40.9 ± 11.3	41.0 ± 11.4	41.7 ± 11.1	41.0 ± 11.1
BMI, mean ± SD, kg/m^2^	42.2 ± 5.2*	42.4 ± 5.4*	41.0 ± 6.0	41.3 ± 6.0
Comorbidity				
Sleep apnea, *n* (%)	609 (11.1)	3589 (11.7)	93 (10.2)	522 (9.7)
Hypertension, *n* (%)	1493 (27.3)	8591 (28.0)	221 (24.2)	1281 (23.7)
Diabetes, *n* (%)	862 (15.8)	4951 (16.2)	111 (12.2)	673 (12.5)
Dyslipidemia, *n* (%)	578 (10.6)	3507 (11.4)	84 (9.2)	477 (8.8)
Dyspepsia/GERD, *n* (%)	660 (12.1)	3747 (12.2)	70 (7.7)	379 (7.0)
Depression, *n* (%)	861 (15.8)	4891 (16.0)	173 (19.0)	959 (17.8)
Concurrent surgery, *n* (%)	180 (3.3)*^1^	1239 (4.0)*^2^	60 (6.6)*^3^	257 (4.8)*^4^

*BMI* body mass index, *SD* standard deviation, *GERD* gastroesophageal reflux disease

*Significant difference with *p*< 0.05

There were no missing data for any of the baseline characteristics variables

^1^More than one procedure/patient possible: adhesiolysis > 10 min 118, abdominal wall hernia 16, cholecystectomy 11, management of hiatal hernia or down-take of previous fundoplication 15, small bowel resection 10, partial gastrectomy 4, gynecological operation 4, liver biopsy 3, splenectomy 1, other 19

^2^Adhesiolysis > 10 min 670, abdominal wall hernia 167, cholecystectomy 126, management of hiatal hernia or down-take of previous fundoplication 138, small bowel resection 71, partial gastrectomy 35, gynecological operation 22, liver biopsy 43, splenectomy 11, other 123

^3^Adhesiolysis > 10 min 25, abdominal wall hernia 6, cholecystectomy 2, management of hiatal hernia or down-take of previous fundoplication 25, partial gastrectomy 1, liver biopsy 2, other 1

^4^Adhesiolysis > 10 min 107, abdominal wall hernia 14, cholecystectomy 4, management of hiatal hernia or down-take of previous fundoplication 127, small bowel resection 1, partial gastrectomy 2, gynecological operation 3, liver biopsy 8, splenectomy 3, other 28

Postoperative complications occurred within 30 days of RYGB surgery in 469 (8.6%) patients during the start-up period after summer closure and in 2533 (8.3%) patients in the control group with 218 (4.0%) versus 1047 (3.4%) fulfilling the criteria for a serious postoperative complication. In the SG group, the overall complication rate was 61 (6.7%) in the group treated after summer closure and 305 (5.7%) in the reference group. The corresponding number of serious postoperative complications were 20 (2.2%) compared to 98 (1.8%) (Tables 2 and 3).

**Table 2:** Incidence of postoperative complications for patients receiving gastric bypass surgery within 4 weeks after summer closure period and those having surgery during the remaining part of the year

	After the summer closure	Remaining part of the year	OR/*B* (95% CI)^a^	*p*
Primary outcome				
Serious complication, *n* (%)	218 (4.0)	1047 (3.4)	1.17 (1.01–1.36)	0.034
Secondary outcomes				
Duration of surgery, mean ± SD, min	68 ± 34	71 ± 34	− 2.1 (− 3.0 to − 1.2)	< 0.001
Hospital readmission, *n* (%)	520 (9.5)	2778 (9.1)	1.04 (0.95–1.15)	0.400
Specified complication				
Leak, *n* (%)	61 (1.1)	331 (1.1)	1.04 (0.79–1.38)	0.756
Bleeding, *n* (%)	109 (2.0)	599 (2.0)	1.02 (0.83–1.25)	0.862
Abscess, *n* (%)	39 (0.7)	240 (0.8)	0.92 (0.65–1.29)	0.618
Small bowel obstruction (%)	78 (1.4)	388 (1.3)	1.12 (0.88–1.43)	0.355
Stricture, *n* (%)	15 (0.3)	69 (0.2)	1.21 (0.69–2.12)	0.504
Deep venous thrombosis, *n* (%)	7 (0.1)	33 (0.1)	1.18 (0.52–2.68)	0.685
Pulmonary complication, *n* (%)	34 (0.6)	174 (0.6)	1.10 (0.76–1.59)	0.619
Cardiovascular complication, *n* (%)	10 (0.2)	37 (0.1)	1.54 (0.76–1.59)	0.231
Wound complication, *n* (%)	65 (1.2)	303 (1.0)	1.21 (0.92–1.58)	0.167
Marginal ulcer, *n* (%)	22 (0.4)	144 (0.5)	0.85 (0.54–1.33)	0.469
Urinary tract infection, *n* (%)	18 (0.3)	87 (0.3)	1.15 (0.69–1.91)	0.590

*OR* odds ratio, *CI* confidence interval, *SD* standard deviation, *N/A* not available.

^a^ORs are based on logistic regression adjusting for sex, age, BMI, comorbidities, concurrent surgery, and year of surgery and are displayed for all adjusted outcomes except for duration of surgery where comparisons of the mean (*B* value) are estimated using linear regression adjusted for the same potential confounders.

**Table 3:** Incidence of postoperative complications for patients receiving sleeve gastrectomy surgery within 4 weeks after summer closure and those having surgery during the remaining part of the year

	After the summer closure	Remaining part of the year	OR/*B* (95% CI)^a^	*p*
Primary outcome				
Serious complication, *n* (%)	20 (2.2)	98 (1.8)	1.17 (0.72–1.91)	0.436
Secondary outcomes				
Duration of surgery, mean ± SD, min	47 ± 24	47 ± 23	0.0 (− 1.5 to 1.5)	0.963
Hospital readmission, *n* (%)	48 (5.3)	260 (4.8)	1.09 (0.79–1.50)	0.587
Specified complication				
Leak, *n* (%)	4 (0.4)	36 (0.7)	0.66 (0.23–1.86)	0.431
Bleeding, *n* (%)	17 (1.9)	68 (1.3)	1.49 (0.87–2.55)	0.145
Abscess, *n* (%)	1 (0.1)	21 (0.4)	0.26 (0.03–1.94)	0.188
Small bowel obstruction (%)	1 (0.1)	4 (0.1)	1.41 (0.87–2.55)	0.761
Stricture, *n* (%)	1 (0.1)	10 (0.2)	0.52 (0.07–4.19)	0.542
Deep venous thrombosis, *n* (%)	3 (0.3)	2 (0.0)	6.90 (1.10–43.29)	0.039
Pulmonary complication, *n* (%)	3 (0.0)	14 (0.3)	0.85 (0.26–2,83)	0.794
Cardiovascular complication, *n* (%)	0 (0.0)	4 (0.1)	NA	0.990
Wound complication, *n* (%)	9 (1.0)	53 (1.0)	1.01 (0.50–2.06)	0.977
Marginal ulcer, *n* (%)	1 (0.1)	4 (0.1)	1.33 (0.14–12.62)	0.804
Urinary tract infection, *n* (%)	2 (0.2)	14 (0.3)	0.80 (0.18–3.59)	0.776

*OR* odds ratio, *CI* confidence interval, *SD* standard deviation, *N/A* not available.

^a^ORs are based on logistic regression adjusting for sex, age, BMI, comorbidities, concurrent surgery, and year of surgery and are displayed for all adjusted outcomes except for duration of surgery where comparisons of the mean (*B* value) are estimated using linear regression adjusted for the same potential confounders.

There was a significant increased risk of serious complications (RR = 1.17; 95% CI 1.01–1.35; *p* = 0.034) for patients who underwent RYGB surgery after summer closure, with similar results after adjustment for age, sex, BMI, comorbidities, and year of surgery (adj-OR = 1.17; 95% CI: 1.01–1.36; *p* = 0.034). The same tendency was seen irrespective of hospital volume. No difference in the risk for serious complications was seen after SG (RR = 1.21; 95% CI 0.75–1.94; *p* = 0.436; adj-OR = 1.17; 95% CI: 0.72–1.91; *p* = 0.519). However, there was an increased risk of deep venous thrombosis (DVT) in the summer closure group after SG surgery (adj-OR = 6.90; 95% CI 1.10–43.29; *p* = 0.039).

Having surgery after summer closure was associated with significantly shorter operative times for RYGB surgery (adjusted mean difference, − 2.1 min; 95% CI: − 3.0 to − 1.2; *p* < 0.001). There was also a shorter duration of postoperative hospital stay after both RYGB (mean ranks difference − 2.8; *p* = 0.05) and SG (mean rank difference − 2.0; *p* = 0.046) in the summer closure group. This would correspond to a measure of effect size for postoperative hospital stay of 0.02% for RYGB and 0.06% for SG.

The sensitivity analysis for RYGB patients was statistically significant regardless of whether all patients lost to follow-up were assumed to have had a serious complication or not. For SG, the analysis was still not statistically significant regardless of the configuration of missing data, that is, no serious complications or only serious complications (Supplementary Tables 1 and 2). No tendency towards increased complications was seen among patients operated after the summer period at centers not closing during summer (Supplementary Table 3).

## Discussion

In the present study, we found that surgery center summer closure was associated with a higher risk of serious complications for patients who underwent laparoscopic RYGB surgery within the first month of start-up. This association remained significant when adjusting for other known risk factors.

Laparoscopic RYGB is a technically advanced procedure and it has been estimated that it takes approximately 100 operations to master the technique [13–15], with wide differences in technical skills between surgeons [16]. However, while many studies of the learning process have been conducted, studies regarding the decay of laparoscopic surgical skills are scarce. For laparoscopic nephrectomy and nephroureterectomy, the time interval between operations influences the outcome in low-volume hospitals. Using a shorter operative time as a measure of surgical skill, deterioration was seen after a 14-day interval between procedures [17]. Many studies investigating surgeons’ increasing proficiency have used decreasing duration of surgery as a measure thereof [13, 14, 18]. In the present study, we found that although the start-up period after summer closure was associated with a higher risk of serious postoperative complications, it was also associated with shorter operation time. Although the shorter operation time in part may be explained by the slightly higher number of concurrent operations in the control group, shorter operation times may therefore not always be proof of higher levels of skill. Sinha et al. found that for general surgery residents performing simulator training, good instrumentation and tissue handling techniques were associated with a more consistent deterioration after an interval of nonuse than the speed at which a task was completed. Adequate task performance was associated with both shorter and longer durations of task completion and the time taken to complete a task showed little association with passing or failing the task. Skills in complex tasks deteriorated more than skills in simple tasks. First-year residents were also more prone to skill decay than upper-level residents [19]. Since the decay of laparoscopic skills in experienced surgeons in high-volume surgery facilities seems almost unexplored, there is room for future research in this field. The technical skills may vary widely even for experienced surgeons and although a sensitivity analysis including hospital volume failed to show major differences in the effect of summer closure, individual surgeons may experience differences in decay from absence from surgery [16].

This study showed that in a large cohort of RYGB patients, the risk of serious complications increased by 17% after summer closure. Since this represents a small increase, starting from a low risk to begin with, having RYGB after the summer vacation may still be considered safe. Nevertheless, in continuous work to reduce complication rates and improve the quality of bariatric surgery, some possible approaches to moderate the effect of intervals of absence may be considered. First, the right type of patient should be selected for the first weeks after a period of absence when the risk of serious complications is slightly increased. Since some risk factors for postoperative complications are already known, it should be possible to select patients with fewer risk factors or to optimize these risk factors before surgery. Second, the surgeon should be teamed with an experienced assistant. Previous research has shown that less experienced first assistants in RYGB and SG surgery increase the 30-day readmission rate and the need for intensive care unit management [20]. Third, with today’s advancement in technology, a great deal of research has focused on the benefits of simulator training [21–24]. It has also been shown that such training is indeed transferable to the operating room [23, 25]. Again, most studies have been conducted on residents and non-experienced laparoscopic surgeons. Hence, it is still unclear whether experienced surgeons would benefit from such training.

In the present study, there was no statistically significant association between summer closure and an increased risk of serious postoperative complications in SG surgery. SG is often viewed as a less advanced procedure than RYGB [26], with slightly shorter learning curve [15], and skill may therefore not be as sensitive to periods of absence. Given that SG has gained ground only in recent years, it is possible that the SG population of this study was too small to attain the statistical power needed for significant results. However, the statistical analysis showed a higher risk of DVT after SG surgery in the summer closure group, which may indicate that SG is not completely insensitive to longer intervals of performed surgeries. While the relation to summer closure remains unclear, SG has been reported to induce a hypercoagulable state [27] as well as a reduced blood flow velocity [28] which may in part explain why an increased risk for this particular complication was seen after SG but not after RYGB, despite the higher overall complication rates after RYGB.

In addition to surgical factors, several important perioperative factors involving the process from preparation before surgery, through perioperative care to the early postoperative period, may also be influenced by summer closure. In Sweden, the adherence to fast-track protocols, such as that presented by the ERAS Society [29], is generally high, but summer closure could at least in theory influence adherence from patients as well as caregivers.

Despite the strengths of nearly complete national coverage, the results of this study must be viewed in light of its limitations. This was a retrospective cohort study based on registry data. Consequently, there may be variables of relevance that were not adequately recorded. One such variable is smoking, which was not a compulsory parameter in SOReg from the beginning. With our low prevalence of smoking (12%) [30], it is unlikely that the number of smokers differed enough between groups to confound the results. Socioeconomic status and surgeons’ experience have also been demonstrated to be relevant variables [15, 31]. Although it is also unlikely that these variables would differ throughout the year, adjusting for smoking, socioeconomic status, and surgeons’ experience as confounders would have been desirable. In the patient selection process, 1699 patients were excluded from the study due to a lack of 30-day follow-up. A comparison of baseline data of patients lost to follow-up and patients included in the study showed similar compositions of age, sex, and BMI in all groups. For both RYGB and SG patients, all comorbidities except depression were slightly less common among patients lost to follow-up. Despite these differences, the total study data loss was < 5%. The robustness of the results was nonetheless tested in a sensitivity analysis. This analysis did not change the statistical significance of the results. Furthermore, despite the lack of seasonal effect for patients operated at centers not closing during summer, we cannot totally exclude a seasonal effect. Finally, the Swedish healthcare system differs from that in other parts of the world, which may limit generalizability. However, the effects of closing during a period of at least 4 weeks are likely to be similar in other healthcare systems.

In conclusion, the results of this study indicate that having RYGB surgery during the start-up period after summer vacation is associated with an increased risk of serious postoperative complications compared to the corresponding risk posed by having this surgery during the rest of the year. No increased risk was found for SG surgery, or for overall complications in either procedure. More research is needed regarding the skill deterioration rate in experienced bariatric surgeons and whether they would benefit from preoperative simulator training after a period of absence. Such training might not completely counteract the effect of longer absences in bariatric surgery, but along with optimizing patient-specific risk factors and teaming up the surgeon with an experienced assistant, it may mitigate the effect of summer closure.

## Supplementary Information

Below is the link to the electronic supplementary material.

Supplementary file1 (DOCX 45 KB)
