# Supplementary material for: The influence of summer closure on serious postoperative complications in bariatric surgery
Source: Langenbecks Arch Surg. 2022 Jun 2;407(7):2769–75. doi: 10.1007/s00423-022-02566-w (PMC9640441; doi:10.1007/s00423-022-02566-w)
Supplement: Supplementary file 1 — Supplementary file1 (DOCX 45 KB) [file 423_2022_2566_MOESM1_ESM.docx]

# Supplementary Tables

| **Supplementary Table 1**. Baseline characteristics of patients lost to follow-up | | | | |
| --- | --- | --- | --- | --- |
|  | Gastric Bypass | | Sleeve Gastrectomy | |
|  | After the summer closure | Remaining part of the year | After the summer closure | Remaining part of the year |
| Number of patients, n | 195 | 978 | 88 | 438 |
| Female sex, n (%) | 145 (74.4) | 713 (72.9) | 74 (84.1) | 344 (78.5) |
| Age at surgery, mean ± SD, yrs | 38.2 ± 10.8 | 37.0 ± 11.2 | 37.8 ± 10.4 | 39.4 ± 10.7 |
| BMI, mean ± SD, kg/m^2^ | 40.4 ± 6.0 | 41.4 ± 5.4 | 37.8 ± 6.1 | 38.9 ± 6.6 |
| Comorbidity |  |  |  |  |
| Sleep apnea, n (%) | 14 (7.2) | 91 (9.3) | 9 (10.2) | 26 (5.9) |
| Hypertension, n (%) | 34 (17.4) | 188 (19.2) | 14 (15.9) | 79 (18.0) |
| Diabetes, n (%) | 15 (7.7) | 109 (11.1) | 5 (5.7) | 41 (9.4) |
| Dyslipidemia, n (%) | 9 (4.6) | 71 (7.3) | 4 (4.5) | 19 (4.3) |
| Dyspepsia/GERD, n (%) | 24 (12.3) | 111 (11.2) | 4 (4.5) | 28 (6.4) |
| Depression, n (%) | 37 (19.0) | 184 (18.8) | 23 (26.1) | 85 (19.4) |
| Concurrent surgery, n (%) | 6 (3.1) | 40 (4.1) | 6 (6.8) | 23 (5.3) |
| BMI = Body Mass Index, SD = Standard deviation, GERD = Gastroesophageal reflux disease | | | | |

| **Supplementary Table 2.** Comparison of patients having surgery within four weeks of summer closure and those having surgery during the remaining part of the year including patients lost to follow-up. | | | | |
| --- | --- | --- | --- | --- |
|  | Gastric Bypass | | Sleeve Gastrectomy | |
|  | OR (95%CI)^c^ | P^c^ | OR (95%CI)^c^ | P^c^ |
| Analyses of primary outcome |  |  |  |  |
| Serious complication, adjusted^a,c^ | 1.17 (1.01 – 1.36) | 0.034 | 1.16 (0.71 – 1.88) | 0.559 |
| Serious complication, adjusted^b,c^ | 1.15 (1.03 – 1.28) | 0.016 | 1.16 (0.93 – 1.44) | 0.193 |
| OR = Odds ratio ; CI = Confidence interval | | | | |
| ^a^ Assuming no patient lost to follow-up had a serious complication | | | | |
| ^b^ Assuming all patients lost to follow-up had a serious complication | | | | |
| ^c^ Multivariable logistic regression adjusting for sex, age, BMI, comorbidities, concurrent surgery and year of surgery | | | | |

| **Supplementary Table 3.** Comparison of patients having surgery during four weeks after summer and those having surgery during the remaining part of the year for patients operated at centers not closing during summer | | | | |
| --- | --- | --- | --- | --- |
|  | Gastric Bypass | | Sleeve Gastrectomy | |
|  | OR (95%CI)^c^ | P | OR (95%CI)^a^ | P^a^ |
| Analyses of primary outcome |  |  |  |  |
| Serious complication, unadjusted | 0.95 (0.61 – 1.48) | 0.823 | 0.31 (0.08 – 1.28) | 0.105 |
| Serious complication, adjusted^a^ | 0.96 (0.62 – 1.49) | 0.861 | 0.32 (0.08 – 1.30) | 0.111 |
| OR = Odds ratio ; CI = Confidence interval  ^a^ Multivariable logistic regression adjusting for sex, age, BMI, comorbidities, concurrent surgery and year of surgery | | | | |
